# Glucocorticoid‐induced osteonecrosis in systemic lupus erythematosus patients

**DOI:** 10.1002/ctm2.526

**Published:** 2021-10-12

**Authors:** Kaichi Kaneko, Hao Chen, Matthew Kaufman, Isaak Sverdlov, Emily M. Stein, Kyung‐Hyun Park‐Min

**Affiliations:** ^1^ Arthritis and Tissue Degeneration Program, David Z. Rosensweig Genomics Research Center Hospital for Special Surgery New York New York 10021 USA; ^2^ Department of Orthopedics Beijing Friendship Hospital Beijing 100050 China; ^3^ Case Western Reserve School of Medicine Cleveland Ohio 44106 USA; ^4^ Tuoro College of Osteopathic Medicine‐New York Campus New York New York 10027 USA; ^5^ Endocrinology Service, Hospital for Special Surgery New York New York USA; ^6^ Metabolic Bone Disease Service, Hospital for Special Surgery New York New York USA; ^7^ Department of Medicine Weill Cornell Medical College New York New York USA; ^8^ BCMB allied program Weill Cornell Graduate School of Medical Sciences New York New York 10021 USA

**Keywords:** glucocorticoid, osteonecrosis, systemic lupus erythematosus

## Abstract

Osteonecrosis (ON) is a complex and multifactorial complication of systemic lupus erythematosus (SLE). ON is a devastating condition that causes severe pain and compromises the quality of life. The prevalence of ON in SLE patients is variable, ranging from 1.7% to 52%. However, the pathophysiology and risk factors for ON in patients with SLE have not yet been fully determined. Several mechanisms for SLE patients’ propensity to develop ON have been proposed. Glucocorticoid is a widely used therapeutic option for SLE patients and high‐dose glucocorticoid therapy in SLE patients is strongly associated with the development of ON. Although the hips and knees are the most commonly affected areas, it may be present at multiple anatomical locations. Clinically, ON often remains undetected until patients feel discomfort and pain at specific sites at which point the process of bone death is already advanced. However, strategies for prevention and options for treatment are limited. Here, we review the epidemiology, risk factors, diagnosis, and treatment options for glucocorticoid‐induced ON, with a specific focus on patients with SLE.

## Graphical abstract

ON is a complex and multifactorial complication of Systemic Lupus Erythematosus (SLE). However, the pathophysiology and risk factors for ON in patients with SLE have not been fully determined yet. Here, we review the epidemiology, risk factors, diagnosis, and treatment options for glucocorticoid‐induced ON, with a specific focus on patients with SLE

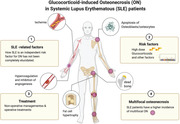

## 1 INTRODUCTION

Osteonecrosis (ON) (also known as avascular necrosis (AVN), atraumatic necrosis, aseptic necrosis, or ischemic necrosis) is a pathologic process of bone cell death that typically affects the middle‐aged population.^1^, ^2^, ^3^ ON is a debilitating disease, causing severe pain and compromised quality of life; ON can occur in several circumstances. Trauma is the most common cause. Atraumatic causes of ON are alcohol and glucocorticoid use, hypercholesterolemia, sickle cell anemia, and autoimmune diseases.^4^, ^5^, ^6^, ^7^, ^8^, ^9^, ^10^ The use of glucocorticoids is one of the most common causes of nontraumatic ON.^9^, ^11^, ^12^, ^13^, ^14^

Osteonecrosis is a well‐known complication of adult and juvenile autoimmune diseases^15^, ^16^ and occurs more frequently in patients with systemic lupus erythematosus (SLE) than in any other rheumatic diseases requiring administration of glucocorticoid.^17^, ^18^ The prevalence of ON in patients with SLE varies between 1.7% and 52%. However, the etiology of ON in SLE patients is incompletely understood. Among several risk factors, glucocorticoid use is strongly associated with the development of ON in patients with SLE.^19^, ^20^, ^21^, ^22^, ^23^ At present, strategies for the prevention and treatment of glucocorticoid‐induced ON are limited, with no effective therapy that can reverse the conditions, primarily because the pathogenesis of ON is poorly understood. In this review, we examine recent evidence of the pathogenesis, risk factors, diagnosis, and treatment options of glucocorticoid‐induced ON in SLE patients.

## 2 THE ACTION OF GLUCOCORTICOIDS

Corticosteroids, which are endogenous hormones derived from cholesterol in the adrenal glands, have been used exogenously as anti‐inflammatory and immunosuppressive agents for decades.^24^ Typically, the human body produces corticosteroids in response to many different stimuli. They are metabolized into glucocorticoids and mineralocorticoids and control many of the body's regulatory processes. Mineralocorticoids such as aldosterone regulate the electrolyte and volume status of the human body. Glucocorticoids, the main actor being cortisol, are endogenously released due to stresses on the body.^25^, ^26^

**HIGHLIGHTS**

1. The use of glucocorticoid treatments is one of the major risk factors for osteonecrosis in SLE patients.
2. The pathogenesis of glucocorticoid‐induced osteonecrosis in SLE patients remains unclear.
3. Glucocorticoid‐mediated changes including changes in angiogenesis, apoptosis of osteocyte, osteoblast, and endothelial cells, as well as adipogenesis and fat hypertrophy, may contribute to the onset of osteonecrosis in SLE patients.

The glucocorticoids from one of the most influential classes of modern medications. Their clinical application began in 1948 when Dr. Philip Showalter Hench used synthetic cortisone for the first time in a patient with rheumatoid arthritis.^14^ Due to their immunosuppressive and anti‐inflammatory effects, glucocorticoids have been widely used to manage autoimmune conditions, inflammatory diseases, allergies, and hematological disorders. These disorders include multiple sclerosis, glomerular disease, Sjögren's disease, sarcoidosis, Graves’ disease, SLE, and rheumatoid arthritis. Glucocorticoids are also used for the local symptomatic treatment of osteoarthritis and tenosynovitis..^27^, ^28^ However, various side effects of long‐term treatment, such as ON and osteoporosis, have been noted.^29^, ^30^, ^31^

Glucocorticoids robustly impact numerous tissues and cell lineages. They induce apoptosis of immune cells such as T cells, basophils, and eosinophils by modifying cytokine release profiles and dampening the immune response and are used to trigger apoptosis in tumor cells as a treatment for multiple myeloma, Hodgkin's lymphoma, and chronic lymphoblastic leukemia.^32^ Glucocorticoids also lead to increased levels of vasoconstriction in peripheral tissues. In addition to the direct impact on immune cells, glucocorticoids lead to changes at the tissue level, leading to side effects such as osteoporosis, euphoria, psychosis, hyperglycemia, and osteonecrosis.^33^

## 3 GLUCOCORTICOID‐INDUCED OSTEONECROSIS

### 3.1 Methods

A comprehensive and systematic search was performed using the MEDLINE/PubMed (U.S. National Library of Medicine, Bethesda, MD). The database was searched in November 2020 from its inception to 2020 for only articles written in or translated into English. A broad combination of Medical Subject Heading (MeSH) terms were used, including “(systemic lupus erythematosus OR SLE) AND reconstruction AND (osteonecrosis OR avascular osteonecrosis OR aseptic necrosis) AND (glucocorticoids OR corticosteroids).”

### 3.2 Epidemiology

Table 1 summarizes the frequency of ON and the risk factors for the development of ON in SLE patients reported by prospective, retrospective, and cohort studies. The association between SLE and ON was first reported in 1960.^19^ Studies have reported incidence rates for ON in SLE patients ranging from 1.7% to 52% (Table 1). This wide range reflects the use of different techniques to diagnose ON (from the less sensitive plain film radiograph to the fairly sensitive MRI), variations in glucocorticoid dosing, and different durations of follow‐up. In addition, the wide variation in prevalence may result from a missed diagnosis of asymptomatic ON.^34^ Data from prospective studies are similarly variable.^25^, ^51^, ^91^, ^93^, ^94^, ^103^, ^114^, ^117^, ^118^, ^119^, ^196^, ^199^ In one study, the incidence of ON increased constantly during each of the first 5 years after a diagnosis of SLE. Only one (0.27%) of 365 SLE patients developed ON in the first year after diagnosis, compared to 0.88% and 3.3% during the second and fourth years after diagnosis.^35^ In another long‐term cohort study, 50% of SLE‐diagnosed patients developed ON within 2 years of diagnosis.^36^ In the largest cohort of SLE patients with symptomatic ON, 234 of 1729 (13.5%) patients with SLE had 581 sites of symptomatic ON. The hips and knees were the most common of these sites and 47% of the patients had multiple sites involved.^37^

**TABLE 1:** Prevalence of osteonecrosis in SLE

					Clinical factors	
Author	Year	Study design	No. of patients	Prevalence (%)	Related factors	Unrelated factors
Dogan et al^40^	2020	Cross‐sectional	127	8.7% (11)	l, u, cc, ff, hh	e, g, o, p, s, w, x
Tsai et al.^98^	2020	Retrospective	1472	2.6% (39)	l, aa	a, l, w, z, ff, gg
Kwon HH et al.^99^	2018	Observational	1219	10.8% (132)	w, z	a, g, l, m, p, r, v, y
Ruiz‐Arruza I et al.^60^	2018	Observational	287	2.4% (7)	Not applicable	Not applicable
Gladman DD et al.^37^	2018	Prospective	1729	13.5% (234)	c, t, u, ff	a, x
Chen HL et al.^113^	2018	Prospective	11288	3.9% (444)	u	Not applicable
Tse SM et al.^46^	2017	Retrospective	275	7.4% (55)	a, t, u, w, cc, dd, ff	e, f, g, h, I, j, n, m, o, p, gg
Sheane BJ et al.^47^	2017	Prospective	173	13.9% (24)	u	s, t
Kuroda et al.^100^	2015	Prospective^#^	78	26.9% (21)	ff	a, b, f, g, j, m, l, s, y
Faezi et al^101^	2015	Retrospective case‐control	oral (314) pulse (351)	21% (66) 11% (39)	a, f, g, m	e, i, l, m, n, p, q, s, hh, ff
Yang et al.^16^	2015	Case‐control	617	6% (37)	l, m, u	a, e, q, s, v, z, aa
Gontero et al.^102^	2015	Observational	158	9.5% (15)	t, cc	a, e, g, i, k, l, m, n, o, q, r, s, cc, gg
Joo et al.^48^	2015	Retrospective	25,358	3.15‐3.42% (8.4‐9.8/1000)	u, y, z, aa, bb, gg	a, b, l, hh
Lee et al.^49^	2014	Retrospective	1051	6.9% (73)	u, w, z, cc	a, b, d, e, f, g, h, i, l, m, n, p, q, r, gg, hh
Ruiz‐Arruza I et al.^115^	2014	Observational	230	1.7% (4)	u	Not applicable
Kunyakham et al.^103^	2012	Retrospective	736	8.8% (65)	d, n	a, b, g, i, n, j, l, m, o, u, z, aa, dd, ff, gg
Sayarlioglu et al.^50^	2012	Retrospective	868	5.6% (49)	a, b, e, f, h, j, m, o, u, w, x, z	g, i, n, l, q, r, ee, ff
Nakamura et al.^104^	2010	Prospective	676	38.5% (260)	a	U
Al Saleh et al.^66^	2010	A cross‐sectional and retrospective case‐control	126	8.7% (11)	g, j, l, o, q, r, t, v, z, aa	e, f, i, n, ff, gg
Sekiya et al.^65^	2010	Prospective^#^	17	29.4% (5)	q, dd	a, b, n, r, u, y
Uea‐areewongsa et al.^105^	2009	Case‐control	186	22% (41)	l, aa	a, d, m, s, u, w, x, z, ff
Fialho et al.^106^	2007	Prospective	46	21.7% (10)	s	e, g, o, q, t, w, z, aa, cc, ff
Prasad et al.^107^	2007	Case‐control	570	11.4% (65)	Not applicable	c, e, i, l, q, s, u, x, y, z, aa, ff, hh
Nagasawa et al.^51^	2005	Prospective^#^	45	44.4% (20)	u, y, ff	a, b, l, m, n, q, v
Oinuma et al.^52^	2001	Prospective^#^	72	44% (32)	u	a, b, s, y
Gladman et al.^36^	2001	Case‐control	744	12.8% (95)	i, u, z,	a, b, c, d, e, l, m, o, q, s, aa, cc, ff
Gladman et al.^53^	2001	Case‐control 70 patients used	744	12.8% (95)	i, u, w, x, z, cc	a, b, c, d, e, g, l, m, n, o, q, r, s, y, ff
Mok et al.^67^	2000	Retrospective	265	4.2% (11)	No association	a, b, c, d, u, w, q
Zonana‐Nacach et al.^54^	2000	Retrospective	539	8.7% (47)	u	w, t, gg
Mok et al.^42^	1998	Case‐control	320	12% (38)	m, q, u, w, z, aa, cc	a, b, d, e, f, l, m, n, o, q, r, s, x, y, gg
Cozen et al.^39^	1998	Follow‐up	488	5% (26)	a, j, l, m, n, gg	c, e, g, i, q, r, u, y, aa
Mont et al.^55^	1997	Cohort	103	30% (31)	o, q, u, cc, ii	c, g, i, l, n, ee, gg
Arranow et al.^41^	1997	Retrospective	66	12% (8)	e, m, u	c, o, q, y
Rascu et al.^108^	1996	Retrospective	280	2.1% (6)	Not applicable	e, i, j, l, m, n, o, q, r, u, w, x, y
Migliaresi et al.^56^	1994	Observational	69	10.14% (7)	u	a, d, q, r, w, y
Nagasawa et al.^34^	1994	Prospective	23	48% (11) 10% (3); syntomic ON	u	a, b, x
Asherson et al.^64^	1993	Retrospective	800	4.6% (37)	Not applicable	Not applicable
Massardo et al.^109^	1992	Retrospective	176	9.7% (17)	v, y, cc	a, b, e, i, j, l, m, n, o
Ono et al.^57^	1992	Prospective	62	14.5% (9)	f, k, l, n, u, ff	e, i, j, m, n, r
Weiner et al.^58^	1989	Follow‐up	172	16.2% (28)	u	e, f, g, i, l, m, n, o, u, cc
Kalla et al.^110^	1986	Retrospective^*^	185	7% (13)	Not applicable	e, w, x, z, ff
Zizic et al.^17^	1985	Prospective	54	52% (28)	u, x	a, b, c, d, e, h, i, l, m, n, q, r, v, cc, ff
Klippel et al.^43^	1979	Retrospective	375	8.3% (31)	u	a, b, e, l, m
Albeles et al.^35^	1978	Follow‐up^*^	365	4.7% (17)	v	a, b, s, u, x
Dimant et al.^111^	1978	Retrospective case‐control	234	9% (22)	Not applicable	a, d, l, o, s, u, w, x
Smith et al.^112^	1976	Retrospective case‐contro	99	7% (7)	Not applicable	a, e, f, i, j, l, m, n, r, u, w, gg
Bergstein et al.^59^	1974	Prospective	35	40 % (14)	u	a, w, x, z
Dubois et al.^18^	1960	Retrospective	400	2.8% (11)	Not applicable	a, b, r

# initial treatment: high‐dose prednisolone, including pulse therapy with methylprednisolone.

* all patients under glucocorticoid therapy.

**Clinical factors**: a. age, b. sex, c. race d. disease duration, e. Raynaud's phenomenon, f. oral ulcers, g. skin involvement h. lymphadenopathy, i. arthritis/ synovitis, j. serositis, k. lung involvement, l. renal involvement, m. neuropsychiatric SLE (NPSLE), n. hematologic involvement, o. vasculitis, p. antiphospholipid syndrome, q. antiphospholipid antibodies, r. seropositive for antibodies, s. SLE disease activity (SLEDAI), t. SLE damage score, u. high‐dose prednisone or prednisolone, v. high initial prednisone or prednisolone dose, w. cumulative dose of prednisone or prednisolone, x. duration of glucocorticoid therapy, y. pulse therapy, z. use of immunosuppressant drugs, aa. hydroxychloroquine, bb. lipid‐lowering agents, cc. Cushingoid body habitus variable, dd. septic arthritis, ee. Sjögren's syndrome, ff. hyperlipidemia, gg. hypertension, hh. osteoporosis, ii. Preeclampsia.

The effect of genetic ancestry and ethnicity on the incidence of ON in SLE patients is poorly characterized. Different cultures have varying genetic risks for developing SLE,^38^ and different ethnicities have varying rates of SLE patients developing ON. At present, specific genes or genetic markers specific to certain ethnicities modifying the risk of ON in SLE patients have not been clearly established.

Most of the studies estimating the prevalence of ON development in SLE patients were conducted in Asia (Table 1). The prevalence of ON in the general population is low. The average estimated number of annual prevalent cases of ON was 28.91 per 100,000 in Korea, based on the Medical Claims Database of the National Health Insurance Corporation.^38^ Although the prevalence of ON in the general population in the United States remains unclear, newly diagnosed patients are estimated to be between 20,000 and 30,000 every year in the United States. Cozen et al reported 7.69% Hispanic, 19.23% Black, and 57.69% white for the incidence of ON in American SLE patients.^39^ A prospective study at the University of Toronto Lupus Clinic has followed SLE patients since 1970. Among 235 ON patients, the distribution of ON was in 67.1% in Caucasian patients, 15.4 % in Black patients, 8.1% in Asian patients, and 9.4% in others ethnicities.^40^ Arranow et al showed that among eight African‐American SLE patients, 75% of whom developed ON,^41^ suggesting that higher disease activity and higher doses of glucocorticoids in African‐American SLE patients may be associated with the higher incidence of ON in patients of African‐American origin. Demographic data of the prevalence of ON in SLE patients needs to be determined to estimate the burden of ON on different ethnicities.

Although ON is a prevalent complication in patients with rheumatic diseases as a whole, the incidence rate of ON is higher in patients with SLE compared to non‐SLE patients (Table 2).^17^, ^18^, ^23^, ^42^ A retrospective study investigated that the frequency of ON as determined from discharge diagnoses of patients with various rheumatic diseases hospitalized at the Clinical Center of the National Institutes of Health from 1962 to 1977.^43^ A prospective MRI study showed that SLE was the most frequent underlying disease for ON patients who received glucocorticoid therapy from 1986 to 2009.^44^ In nationwide epidemiologic surveys conducted in Japan and Sweden in the 2000s,^3^, ^45^ ON occurred more frequently in patients with SLE than in any other rheumatic diseases requiring the administration of glucocorticoids. Therefore, glucocorticoids maybe not the only risk factor for the incidence of ON in SLE patients.

**TABLE 2:** Prevalence of osteonecrosis in rheumatic diseases

Underlying diseases	Prevalence (%)
Systemic lupus erythematosus	1.7‐52
Rheumatoid arthritis	0.4‐4.8
Polymyositis/dermatomyositis	0.1‐4.9
Granulomatosis with polyangiitis	3.7
Polymyalgia rheumatica	3.3
Mixed connective tissue disease	2.6
Polyarteritis nodosa	2.1
Giant cell arteritis	1.2
Sjögren's syndrome	0.9‐1.1
Behçet's disease	0.4
Ankylosing spondylitis	0.4

### 3.3 Pathogenesis

**FIGURE 1:**
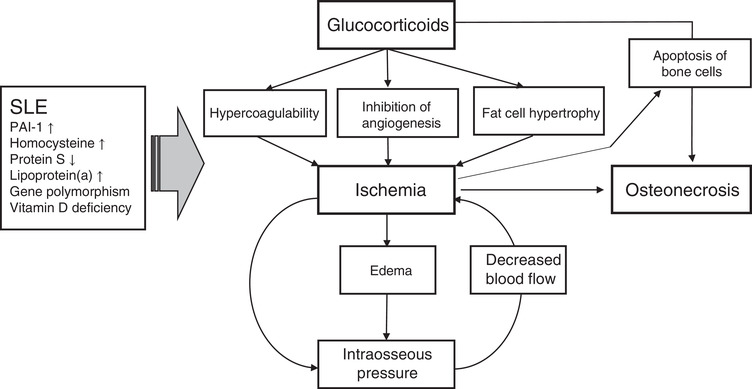
Pathophysiology of glucocorticoid‐induced osteonecrosis (ON) in systemic lupus erythematous (SLE) patients. The pathogenesis of GC‐induced ON in SLE patients remains unclear. Glucocorticoids (GCs) are steroid hormones that can modulate many aspects of cell biology; different GC‐mediated mechanisms, including hypercoagulability, inhibition of angiogenesis, fat cell hypertrophy, and apoptosis of bone cells, have been postulated for the onset of ON. It has also been suggested that GCs may cause ischemic ON through edema‐mediated increases in intraosseous pressure and decreased blood flow. Apoptosis of osteoblasts/osteocytes can be accelerated by ischemia. Although the various pathophysiology of SLE can contribute to the pathogenesis of ON, the contribution of individual SLE‐related factors to the development of ON has yet to be fully elucidated.

Osteonecrosis is a well‐known comorbidity of patients with SLE.^19^, ^20^, ^21^, ^22^, ^42^ The use of glucocorticoid therapy is a major risk factor for the incidence of ON in SLE patients.^16^, ^17^, ^34^, ^35^, ^37^, ^40^, ^41^, ^43^, ^46^, ^47^, ^48^, ^49^, ^50^, ^51^, ^52^, ^53^, ^54^, ^55^, ^56^, ^57^, ^58^, ^59^, ^60^ Multiple factors have been implicated in the pathogenesis of ON in patients who use glucocorticoids.^9^, ^11^, ^12^, ^13^ As glucocorticoids can induce changes in angiogenesis, intravascular coagulation, apoptosis of bone cells, and fat hypertrophy, glucocorticoid‐mediated alterations may contribute to bone ischemia and necrosis by both intra‐ and extraluminal obliteration. Figure 1 summarizes the current understanding of the pathogenesis of ON in SLE patients, which is likely the combined result of multiple factors. However, the pathogenesis of ON in SLE patients remains unclear.

#### 3.3.1 Hypercoagulability and inhibition of angiogenesis

Coagulation and congestion lead to decreased blood flow and oxygen delivery through the microvasculature, leading to an increased risk of developing ON. A genetic association between inherited thrombophilia, hypofibrinolysis, and ON has been established.^61^ It has been suggested that hypofibrinolysis results in increased clot formation, decreased blood flow, and a hypoxic environment within the bone structure, which may lead to ON. The dysregulation of coagulation and complement‐related pathways has been found in patients with SLE and contributes to SLE disease activity.^62^ Furthermore, the presence of antiphospholipid antibodies in SLE can lead to a hypercoagulable state, which is believed to contribute to ON in SLE by promoting intravascular coagulation and congestion.^63^ However, the association between ON and antiphospholipid antibodies in SLE patients remains controversial. The role of antiphospholipid antibodies in the occurrence of ON has been found in only a few studies,^23^, ^55^, ^64^, ^65^, ^66^ while others have not exhibited any association with ON.^67^

Hypercoagulability can be compounded by the glucocorticoid‐mediated inhibition of angiogenesis.^68^ Glucocorticoids can directly injure endothelial cells,^69^ enhance hypercoagulability,^70^ and inhibit angiogenesis by inducing apoptosis of bone marrow endothelial cells (BMECs).^71^ Glucocorticoids can inhibit angiogenesis by diminishing proliferating capillary haemangiomas,^72^ tube‐like structure formation,^73^ new vessel formation,^74^ and angiogenic factor generation.^75^ This decrease in normal angiogenesis, caused by glucocorticoids, in the femoral head and bone tissue can lead to ON. Atsumi et al found that all patients with unilateral glucocorticoid‐induced ON had abnormal superior retinacular arteries, small arterial penetration, and interruption of revascularization in the contralateral normal hips.^76^ Glucocorticoids, in combination with active inflammation or surgery, can also contribute to a hypercoagulable state.^77^ ON does not result from a single episode of impaired blood supply, but from a chronic blockade of microcirculation,^78^ the alteration of BMECs,^79^ and transcriptomic changes in bone microvascular endothelial cells.^80^, ^81^ Apoptotic endothelial cell death has been hypothesized to function as a mechanism for the capillary rarefaction in glucocorticoid‐mediated hypertension.^82^ In turn, glucocorticoid‐induced hypertension in the femoral head disturbs the blood flow in the femoral head vessels and aborts the repair process.^12^

#### 3.3.2 Apoptosis of osteoblasts and osteocytes

The apoptosis of osteoblasts or osteocytes plays a crucial role in the pathogenesis of ON.^83^, ^84^, ^85^ Increased apoptotic osteoblasts or osteocytes have been observed in mice and humans under glucocorticoid therapy.^86^ It has been reported that apoptosis of osteocytes in the femoral head increases during the development of ON, and the percentage of apoptotic cells is significantly increased in the femoral heads of patients with glucocorticoid‐induced ON. However, apoptotic bone cells are notably rare in bone from patients with alcohol‐induced ON^87^, ^88^ and absent in those with trauma and sickle cell‐induced ON. Apoptosis of the bone cells can result from increased inducible nitric oxide synthase (iNOS) and cytochrome C expression^89^ and aberrant metabolites in the synovial fluids.^90^ Although the death of bone cells is observed in the bone of patients with ON, the correlation between the apoptosis of bone cells and SLE‐associated ON has not been clearly established.

#### 3.3.3 Fat cell hypertrophy

Fat cell hypertrophy and fat emboli have been detected in rabbits after the exposure to high‐dose cortisone for 5 months.^91^ Fat emboli can activate the complement pathway, deposit immune complexes, and increase intravascular coagulation,^92^ leading to ON. Glucocorticoids can skew the differentiation of bone marrow stem cells into adipocytes cells by upregulating adipocyte transcription factor gene expression and downregulating the gene expression of osteoblast transcription factors.^91^ Glucocorticoids increase lipid deposition leading to larger numbers of fat cells when compared to normal ratios of parenchyma in the mesenchymal stem cells of ON patients.^93^

In SLE patients, high‐dose glucocorticoids result in an early and rapid drop in bone mass with a marked increase in body fat.^94^ These patients also have elevated levels of serum adiponectin^95^ and postmenopausal SLE women were found to have altered body composition and increased visceral adipose tissue.^96^ These factors, however, were not correlated with glucocorticoid dose. Intraosseous pressure is increased by the growth of fat cells in the intraosseous compartment and results in sinusoidal compression, leading to decreased perfusion of the bone, which may cause ON.^97^

## 4 RISK FACTORS FOR THE DEVELOPMENT OF ON IN PATIENTS WITH SLE

Glucocorticoid exposure is a well‐recognized risk factor for ON in SLE patients (Table 1).^15^, ^16^, ^17^, ^34^, ^35^, ^39^, ^40^, ^41^, ^43^, ^46^, ^47^, ^48^, ^49^, ^50^, ^51^, ^52^, ^54^, ^55^, ^56^, ^57^, ^58^, ^59^, ^64^, ^65^, ^66^, ^67^, ^98^, ^99^, ^100^, ^101^, ^102^, ^103^, ^104^, ^105^, ^106^, ^107^, ^108^, ^109^, ^110^, ^111^, ^112^, ^113^ In particular, the correlation between ON and the doses of glucocorticoid (GC) therapy has been identified in many studies (Table 1) and higher doses of GC therapy have been cited as one of the strongest predictive factors for developing ON. Twenty‐four of 49 studies shown in Table 1 found a significant correlation between the dose of GC therapy and the occurrence of ON in patients with SLE. A meta‐analysis suggests that high‐dose glucocorticoid therapy may increase the risk of ON by as much as 10 times.^22^ Daily doses greater than 40 mg are associated with increased risk, with the incidence rate climbing by approximately 3.6% for every 10 mg increase.^21^ According to a 12‐year longitudinal study, doses greater than 10 mg in combination with high‐intensity GC use (≧80%) are associated with increased risk of osteonecrosis.^113^ A recent large cohort study that has followed SLE patients since 1970 utilized multivariate analysis to identify GC dose as a major predicative factor for the incidence of symptomatic ON.^37^ However, the association of the duration or route of GC therapy with the incidence of ON remains contentious. Intravenous pulses of methylprednisolone are often used to treat severe symptoms in SLE patients and have significantly higher immunosuppressive and anti‐inflammatory effects compared to other delivery methods for glucocorticoids.^114^ Although high‐dose intravenous pulses of methylprednisolone resulted in higher cumulative doses of GCs, its association with the occurrence of ON has conflicting evidence. An MRI study strongly linked GC pulse therapy with the early development of silent ON in SLE patients,^51^ and Massardo et al closely related GC pulse therapy to high‐dose intravenous pulses of methylprednisolone.^109^ By contrast, several studies have also shown that GC pulse therapy is uncorrelated with ON.^17^, ^23^, ^53^, ^54^, ^56^, ^63^ A nationwide epidemiologic study in South Korea demonstrated that the route of glucocorticoid therapy does not affect SLE patient outcomes and oral and intravenous high‐dose glucocorticoids carry equal risk.^48^ Consistently, in recent investigations, GC‐related damages including ON were associated with high cumulative doses and high intensity of oral GCs and lower doses of GC use significantly diminished GC‐induced damages even in the presence of methylprednisolone use.^60^, ^113^, ^115^ These studies also suggest that pulse methylprednisolone may not be an independent association factor for AVN.^50^, ^54^, ^115^ The duration of GC therapy was significantly longer in the AVN group compared to the control group.^17^, ^50^ However, Oinuma et al found that all osteonecrotic legions were detected very early, within 5 months after starting high‐dose glucocorticoid therapy in 72 patients with SLE.^52^ This study suggests that silent ON can occur early after the onset of glucocorticoid therapy. During long‐term, low‐dose glucocorticoid therapy, there were no new cases of ON^116^ and spontaneous repairs were even observed.^117^ Although the etiology of ON in SLE patients is multifactorial, high‐dose GC use remains a key risk factor for ON.

**FIGURE 2:**
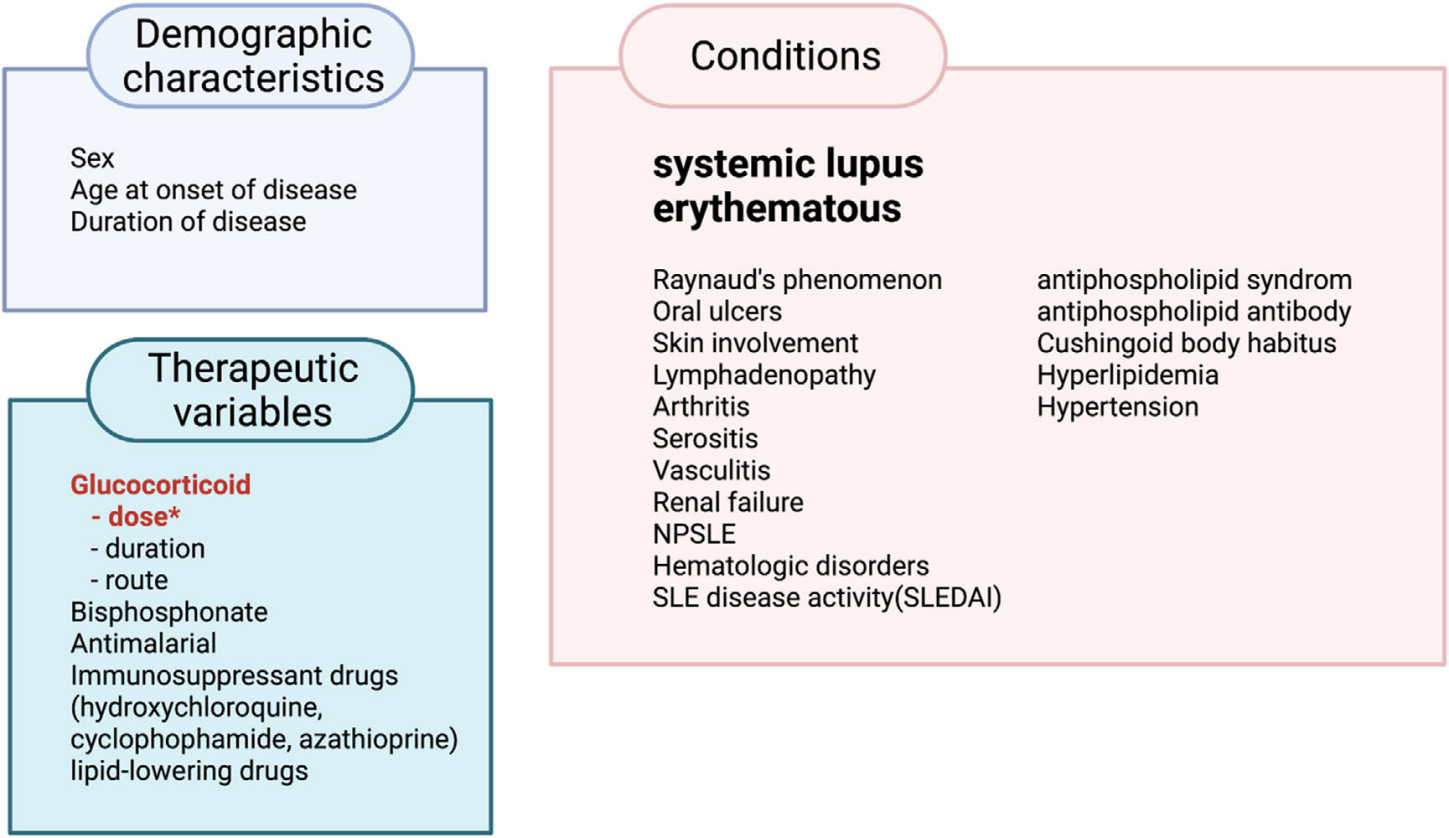
Risk factors of glucocorticoid‐induced osteonecrosis. Many studies have shown that high‐dose GCs are a major risk factor for the onset of ON in systemic lupus erythematous (SLE) patients (red*). The association of the route and duration of GC therapy with the development of ON is still controversial. Risk factors for ON are not limited to GC therapy. Various clinical factors, laboratory factors, and medications have been suggested to be correlated to the onset of ON. Many clinical manifestations of SLE patients has also been shown to impact the incidence of ON in some reports, while others have not exhibited any association with ON

Studies show that SLE patients are at increased risk of developing ON as compared with patients who have other autoimmune conditions and receive similar doses of glucocorticoids. The risk factors for ON are not limited to glucocorticoids.^19^ Many studies have shown that ON in SLE patients is associated with various clinical factors, such as age, sex, disease duration, symptoms of SLE, laboratory factors, medications, and complications of SLE (Table 1 and Figure 2). Although the age at the time of glucocorticoid therapy is considered as a risk factor for ON,^104^ most adult studies identified that age is not a key risk factor for ON in SLE patients. The incidence of ON is relatively low in growing individuals but rapidly increases in adolescents and adults.^44^ There is a report showing a lower incidence of glucocorticoid‐induced ON in pediatric SLE patients (<15 years old) than in adult SLE patients (>20 years old).^104^ Another dynamic MRI study identified higher blood supply in the growth plate of the femoral neck in pediatric SLE patients than adult SLE patients after glucocorticoid therapy.^118^

ONOsteonecrosis also has an increased incidence in SLE patients who have not been taking high‐dose glucocorticoids.^15^ How SLE is an independent risk factor for ON has not yet been elucidated. Disease‐related factors, including Raynaud's phenomenon and vasculitis, also determine ON risk among patients with SLE.^51^, ^65^, ^100^, ^102^, ^106^, ^110^ A long‐term follow‐up study found that SLE recurrence is a risk factor for ON. Among 106 SLE patients, SLE recurrence occurred in 131 joints. The mean time from SLE recurrence to the appearance of new osteonecrotic lesions was 6.2 months. In one single‐center retrospective study with 88 consecutive SLE patients, a high antiphospholipid score was a risk factor for idiopathic ON in SLE patients.^119^ There are conflicting results for the association of antiphospholipid antibodies with ON (Table 1). Some studies showed a positive correlation,^20^, ^66^, ^101^ while many other studies showed no significant correlation with the incidence of ON in SLE patients.^39^, ^46^, ^49^, ^55^, ^58^, ^99^, ^100^, ^102^, ^103^, ^106^ A higher frequency of Cushingoid body habitus showed an association with the incidence of ON in SLE patients.^40^, ^46^, ^49^, ^55^, ^109^

It is well known that glucocorticoids induce iatrogenic metabolic syndrome. Due to this, hyperlipidemia is considered to be an important risk factor for glucocorticoid‐induced ON in SLE. Twelve prospective studies of glucocorticoid‐induced ON in SLE patients have been conducted (Table 1). In addition to a high dosage of glucocorticoids, hyperlipidemia was commonly seen as a risk factor for ON in prospective studies. One prospective study of 78 SLE patients treated with high‐dose prednisolone, showed a correlation between elevated levels of triglyceride and an increased incidence of ON.^100^ In addition, the chronic systemic inflammation or high levels of oxidized LDLs in SLE patients can affect the skeletal phenotype by indirectly influencing bone cells.^120^ ON can be caused by the partial or total disruption of blood flow to the femoral head, and SLE patients tend to develop ON through a similar mechanism. SLE patients with ON are more likely to have hypofibrinolytic 4G polymorphism of the plasminogen activator inhibitor‐1 gene, methylenetetrahydrofolate reductase gene mutation with a higher concentration of homocysteine, low protein S values, and higher lipoprotein(a) levels than controls.^50^ SLE patients are invariably encouraged to avoid sunshine exposure, as this increases the risk of vitamin D deficiency. One juvenile SLE study reported that vitamin D deficiency was significantly associated with subjects who had or developed ON.^23^ However, the contribution of the autoimmune pathophysiology in SLE disease to the development of ON has not yet been fully clarified.

Disease‐modifying anti‐rheumatic drugs (DMARDs), such as hydroxychloroquine, azathioprine, and sulfasalazine, are becoming more prevalent in the management of autoimmune conditions. However, there are a few studies investigating the impact of DMARDs on rates of developing ON in SLE patients. Six of the eight studies shown in Table 1 reported an inverse correlation between the use of hydroxycholoroquine and ON development, although this difference was not statistically significant. A recent retrospective study of the Taiwanese population involved 1472 children newly diagnosed with SLE, and 1364 of these patients had DMARDs as a part of their management.^98^ Although the patients did not have statistically different usages of DMARD's between the ON and non‐ON groups, the cumulative duration of hydrocychloroquine use was significantly correlated with ON. Association between the use of biologic DMARDs, including Bellimumab^121^ and Rituximab, and ON in SLE patients has not been established yet, although Rituximab, an anti‐CD20 antibody, has been suggested as a cause of medication‐related ON of the jaw.^122^ The use of DMARDs may result in the use of lower doses of glucocorticoids in SLE patients, which may affect the incidence of ON in SLE patients. Overall, the current research is insufficient to understand the impact of DMARD's on ON in SLE patients and further research needs to be conducted to fully understand the correlation between DMARDs and ON.

Recent studies have also revealed some new genetic risk factors for ON in SLE patients. Several association studies using targeted next‐generation sequencing technologies identified single nucleotide variations (SNVs) for developing ON in SLE patients (Table 3). Kim et al. have identified that Asp258Asp (exon 6: rs1549758) and Glu298Asp (exon 7: G895T: rs1799983) polymorphisms in the nitric oxide synthase 3 (NOS3) gene may be related to ON susceptibility in SLE patients under the recessive model.^123^ A case‐control study demonstrated that rs3813946 in the 5′‐UTR, rs311306 in intron 1, and the rs17615 in exon 10 of the CR2 (complement receptor type 2, complement C3d Receptor 2) gene.^124^ A recent study has also identified that SNPs for ON risk in SLE patients include NOS3 (exon 6: c.814G > A, p.E272K), Collagen Type II Alpha 1 Chain (COL2A1, c.3508G > A, rs41263847: exon 29: c.1913C > T: p.T638I, exon 28: c.1706C > T: p.T569I, and rs371445823: exon 8: c.580G > A: p.A194T, exon 7: c.373G > A: p.A125T),^125^ and CR2 (rs45573035: exon 2: c.200C > G: p.T67S).^125^ Most of the association studies were conducted in Asia and used a small sample size. Since ethnic difference has been identified across many association studies of genetic polymorphisms in ON, further studies for evaluating these SNPs in other ethnicities are needed. More elaborately designed and larger‐scale studies may clarify more SNPs and their functions in the ON susceptibility of SLE patients. The contribution of genetic predisposition to SLE etiology has been increasingly appreciated, and over 100 susceptibility loci for SLE risk have been identified.^126^, ^127^ However, the genetic predispositions associated with ON susceptibility in SLE patients, including ancestry effects, have not yet been demonstrated. Polymorphism of T786C NOS3 in the promoter was associated with idiopathic ON^128^ but not with ON in SLE patients,^123^ suggesting the influence of different ethnic groups on genetic variation. Investigation of the prevalence of genetic risk factors associated with ON susceptibility in both the general population and in patients of varying ancestries, is a consideration for a future study. To summarize, understanding the impact of genetic variation may provide new insight into ON and ultimately lead to new treatment methods.

**TABLE 3:** Single nucleotide variant in NOS3, COL2A1, and CR2

Gene name	Genotype, rs#	Location	References
NOS3 (nitric oxide synthase 3)	rs1549758	exon 6	^123^
	G895T: rs1799983	exon 7	^123^
	c.814G > A: p.E272K,	exon6	^125^
COL2A1 (Collagen type II alpha‐1 gene)	c.1913C > T: rs41263847: p.T638I	exon 29	^125^
	c.1706C > T: p.T569I	exon 28	^125^
	c.580G > A: rs371445823: p.A194T	exon 8	^125^
	c.373G > A: p.A125T	exon 7	^125^
CR2 (Complement receptor type 2)	rs3813946	5′‐UTR	^124^
	rs311306	intron 1	^124^
	G639A: rs17615	exon 10	^124^
	c.200C > G: rs45573035: p.T67S	exon 2	^125^

## 5 GLUCOCORTICOID‐INDUCED OSTEONECROSIS ANIMAL MODELS

Animal models of ON of the femoral head (ONFH) are indispensable to the understanding of the mechanism, treatment, and prevention modalities for ON of the femoral head. Different animal models for glucocorticoid‐induced ON have been generated. Of all experimental models, rabbits are most commonly used to establish glucocorticoid‐induced ON.^129^ However, these studies were not able to develop joint collapse, which was mainly explained by the lower mechanical loading onto the weight‐bearing joints. Administration of a single glucocorticoid proved to be an efficient way to induce ON in rabbits or mice,^130^, ^131^ resulting in the incidence of ON with this methodology ranging from 10% to 43%. It is necessary to develop an SLE mouse model with a high incidence of glucocorticoid‐induced ON to elucidate the prevention and treatment efficiency of the pharmacological therapy strategies.

### 5.1 Clinical course and diagnosis

#### 5.1.1 Pain

The earliest clinical symptom of ON is bone pain that limits motion, is persistent, and is aggravated by weight‐bearing and activity.^132^ When patients have this persistent bone pain, providers typically order imaging studies to further understand the etiology of this bone pain.

#### 5.1.2 Radiology

Conventional radiography is the first‐line investigation for the diagnosis of ON.^13^ ON is usually diagnosed by X‐ray and magnetic resonance imaging (MRI); however, imaging studies at present do not have the sensitivity to screen for ON at earlier and more manageable stages. Scanning with MRI is the most sensitive modality for diagnosing ON, although it is costly.^133^ The Canadian and American guidelines for SLE recommend radiography as the initial imaging modality for patients suspected of ON, and magnetic resonance imaging (MRI) or single‐photon emission computed tomography (SPECT) if X‐rays are not informative^134^ ^,^.^135^ Whole‐body short‐TI inversion recovery MRI (STIR MRI) has shown promising results in early studies, suggesting that it may become one of the most sensitive and rapid tools for detecting ON lesions at early stages. One study used whole‐body STIR MRI to evaluate ON in 40 adolescents with SLE who received glucocorticoid treatment; there they found seven patients (17.5%) with ON in the knee, hip, and ankle and 37 ON lesions overall.^136^ In addition, MRI can quantify the area of ON.^137^ A 20‐year retrospective study showed that among 30 SLE patients, more than half of those treated with glucocorticoids were already in late‐stage ON when clinical manifestations arose.^138^ However, at present, there are no universal screening guidelines for ON to catch disease at an early stage when conservative and minimally invasive treatments are indicated. The routine MRI screening of SLE patients may facilitate the early detection of ON; however, other constraints, such as financial considerations and resource scarcity, need to be evaluated.

### 5.2 Staging

Classically, ON presents in the femoral head, but it can lead to isolated lesions in other locations such as the jaw or knees. It can also be multifocal. Detection and classification, using one of the four classification systems (Ficat, UPenn, ARCO, and Japanese Orthopedic Association), are crucial to choosing the appropriate treatment modality. However, there are still no guidelines on the imaging screening of ON in SLE patients.

#### 5.2.1 Classification

The classification system of ONFH is crucial to deciding the appropriate clinical intervention. Four classification systems are used to classify ONFH, regardless of etiology: the Ficat Classification (used most commonly), the University of Pennsylvania System, the Association Research Circulation Osseous (ARCO) System, and the Japanese Orthopedic Association system (Tables 4, 5, 6, 7, 8).^139^ Notably, the Association Research Circulation Osseous (ARCO) classification system has undergone revisions recently to eliminate stage 0 and divide stage III into stages IIIA (femoral head depression less than or equal to 2 mm) and stage IIIB (femoral head depression more than 2 mm; see Table 8).^140^ Future directions of classification may include combining findings from digital subtraction angiography (DSA) and MRI to establish the staging of intraosseous circulation obstruction based on the blood supply status of the femoral head.^141^ This staging is based on changes in blood circulation, which can better provide guidance for the treatment strategy to preserve the femoral head preservation, especially in young patients.^141^

**TABLE 4:** Ficat Classification

Stage	Radiographic Findings
1	None (only evident on magnetic resonance images)
2	Diffuse sclerosis, cysts (visualized on radiographs)
3	Subchondral fracture (crescent sign; with or without head collapse)
4	Femoral head collapse, acetabular involvement, and joint destruction (osteoarthritis)

**TABLE 5:** Classification System of the University of Pennsylvania (Steinberg)

Stage	Criteria
0	Normal radiograph, bone scan, and magnetic resonance images
I	Normal radiograph. Abnormal bone scan and/or magnetic resonance images A: Mild (< 15% of femoral head affected) B: Moderate (15% to 30% of femoral head affected) C: Severe (> 30% of femoral head affected)
II	Cystic and sclerotic changes in femoral head A: Mild (< 15% of femoral head affected) B: Moderate (15% to 30% of femoral head affected) C: Severe (> 30% of femoral head affected)
III	Subchondral collapse without flattening (crescent sign) A: Mild (< 15% of articular surface) B: Moderate (15% to 30% of articular surface) C: Severe (> 30% of articular surface)
IV	Flattening of femoral head A: Mild (< 15% of surface and < 2 mm of depression) B: Moderate (15% to 30% of surface and 2 to 4 mm of depression) C: Severe (> 30% of surface and > 4 mm of depression)
V	Joint narrowing or acetabular changes A: Mild B: Moderate C: Severe
VI	Advanced degenerative changes

**TABLE 6:** The 2019 Revised ARCO Staging Criteria

ARCO Stage	Image Findings	Description
I	X‐ray normal, MRI abnormal	A band lesion of low signal intensity around the necrotic area is seen on MRI. A cold spot is seen on bone scan. No changes are seen on plain radiographs.
II	X‐ray abnormal, MRI abnormal	Osteosclerosis, focal osteoporosis, or cystic changes are seen in the femoral head on plain radiographs or CT scan. Still there is no evidence of subchondral fracture, fracture in the necrotic portion, or flattening of the femoral head.
III	Subchondral fracture on X‐ray or CT	Subchondral fracture, fracture in the necrotic portion, and/or flattening of the femoral head is seen on plain radiography or CT scan.
IIIA (early)		Femoral head depression ≤2 mm
IIIB (late)		Femoral head depression > 2 mm
IV	X‐ray osteoarthritis	Osteoarthritis of the hip joint with joint space narrowing, acetabular changes, and destruction are seen on plain radiographs

**TABLE 7:** Radiographic Classification System of the Japanese Orthopaedic Association

Stage	Finding
1	Demarcation line, subdivided by relationship to weight‐bearing area (from medial to lateral) 1A 1B 1C
2	Early flattening WITHOUT demarcation line around necrotic area
3	Cystic lesions, subdivided by site in the femoral head 3A (medial) 3B (lateral)

**TABLE 8:** Comparison of Classification System of the ARCO 1994 and 2019

Radiologic Findings	ARCO Stage in 1994	ARCO Stage in 2019
Preclinical and preradiographic	0	
Evident change on MRI	I	I
Evident change on X‐ray	II	II
Subchondral fracture	III	
Head collapse 2 mm		IIIA
Head collapse > 2 mm		IIIB
Joint space narrowing or acetabular changes	IV	IV

#### 5.2.2 Musculoskeletal manifestations of SLE

Glucocorticoid‐induced ON frequently develops at the femoral head in the hip^9^ but many other sites, such as the knee, shoulder, ankle, and hand can also be affected simultaneously.^142^, ^143^, ^144^ Multifocal ON, a rare variant appearing in only 3.3% of all patients with ON, is defined as the presence of osteonecrotic lesions in three or more separate anatomical sites.^144^ Intriguingly, SLE patients receiving long‐term glucocorticoid treatment have a higher incidence of multifocal ON.^37^, ^145^, ^146^ A multicenter study of patients with multifocal ON reported that 38% (38 of 101) of the multifocal ON patients had a previous SLE diagnosis.^146^ Of these patients, chronic exposure to glucocorticoid therapy was the most common risk factor (91%). All 101 patients with multifocal disease had femoral head involvement. The most common additional sites were in the knee (96%), shoulder (80%), and ankle (44%) with seven other joints also being implicated. The clinical manifestations associated with multifocal ON appeared to be similar to those with non‐multifocal involvement. Patients with SLE who develop multifocal ON tend to be younger, have several SLE clinical manifestations and serological abnormalities, and most have been exposed to glucocorticoid and immunosuppressive agents.^147^, ^148^

### 5.3 Treatment

**FIGURE 3:**
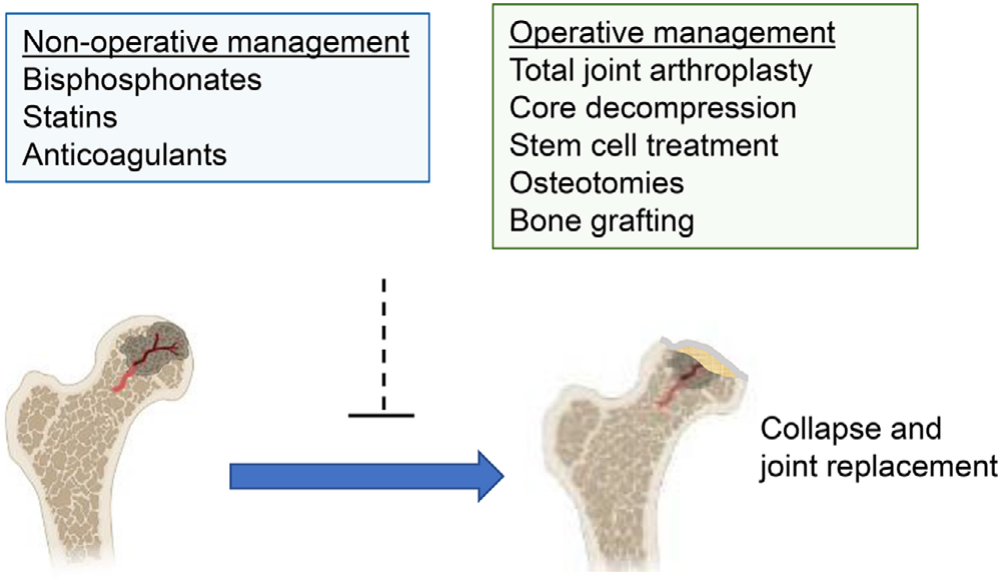
The treatment of glucocorticoid‐induced osteonecrosis. The treatment of GC‐induced ON consists of two approaches: non‐operative management and surgical management. Pharmacological treatments such as bisphosphonates, statins, and anticoagulants have been used. There are surgical interventions for more advanced stages of ON such as core decompression with or without bone grafting, rotational osteotomy, and hip replacement. Although there are no accepted treatments to cure osteonecrosis, the progression to collapse and joint replacement may be prevented if ON is diagnosed early

The treatment of glucocorticoid‐induced ON includes non‐operative management and surgical approaches (Figure 3). Treatments range from ON prophylactic medications such as bisphosphonates, statins, and anticoagulants, which show mixed efficacy, to surgical interventions for more advanced diseases such as core decompression with and without bone grafting, rotational osteotomy, and hip replacement. The choice of treatments is dependent on the stage of the ON, the size of the lesion, the age of the patient, and the patient's co‐morbidity.

#### 5.3.1 Non‐operative management

There is no uniformly accepted treatment for ON.^149^ Various pharmacological agents, such as bisphosphonates, statins, and anticoagulants, have been used to promote viable bone growth within necrotic lesions and alleviate pain and the progression of ON. The effects of these drugs are limited, and additional research is needed to establish the efficacy of these individual agents before routine use of any of them is recommended. However, reports analyzing the effect of non‐operative management for glucocorticoid‐induced ON in SLE patients are limited. Further studies are needed in order to clarify the effects of these treatments on the incidence of ON in SLE patients,

##### Bisphosphonates

Open‐label studies show that bisphosphonates have shown efficacy in preventing disease progression in ON and in delaying the progression to surgery,^149^, ^150^ although the efficacy of alendronate, a bisphosphonate, in ON is still controversial. A study by Agarwala et al of 60 ON patients, 10 of whom had SLE, showed that alendronate reduces pain, improves function, and delays ON progression.^151^ One study analyzed the time from a diagnosis of ON to hip replacement as a marker for the ability of alendronate to minimize disease progression. In the placebo control group, seven hips had a total hip arthroplasty (THA) in the first 12 months and 12 hips by 18 months. In the alendronate group, only six patients progressed to THA and the proportion of hips that developed collapse was also significantly smaller. In contrast, another two‐year, multicenter, prospective, randomized, double‐blind, placebo‐controlled trial study stated that there was no statistical difference in progression to THA between placebo (13%) and bisphosphonate (15%) usage.^152^ Overall, the evidence of the effects of bisphosphonates on glucocorticoid‐induced ON with SLE is mixed, and further studies are needed to evaluate the effect of various bisphosphonates on glucocorticoid‐induced ON for SLE patients.

##### Statins

Hyperlipidemia has also been associated with ON.^8^ Lipid‐lowering agents such as atorvastatin and Lipitor have been tested as treatments for ON in a randomized controlled clinical trial^153^ and in animal models.^154^ Lipid‐lowering agents are thought to prevent ON by reducing the differentiation of marrow pluripotent cells into fat cells, which may lead to increased intraosseous pressure. One database review of 284 patients found a reduced incidence of glucocorticoid‐induced ON among individuals who were on a statin before the initiation of glucocorticoid treatment.^155^ Only 1% of patients develop ON over the 5‐year period. Prospective clinical studies are needed to clarify the effects of statins on glucocorticoid‐induced ON with SLE.

##### Anticoagulants

Anticoagulants, such as warfarin or enoxaparin, have not significantly prevented the progression of disease in ON patients with SLE despite a decreasing tendency to progress to THA.^156^ One study evaluated 60 patients with SLE receiving high‐dose glucocorticoid treatment, treating about half of the patients for anticoagulation with warfarin.^157^ Although the differences were not statistically significant, fewer patients in the warfarin group developed ON compared with the control group (21% versus 33%). Similarly, a prospective study of 35 hips with Ficat stage 1 and two cases of ON with thrombophilia reported that with enoxaparin (60 mg/day for three months) only 20% of patients showed progression of the disease over a 2‐year period.^158^ Another prospective study using warfarin in SLE showed no statistically significant effect of anticoagulant therapy on the prevention of glucocorticoid‐induced ON.^159^ To summarize, the data suggest that anticoagulants did not significantly alter the progression nor affect the prevention of ON.

#### 5.3.2 Surgical Management

Total hip arthroplasty has become most the effective treatment for ON, while non‐arthroplasty treatment options for the management of ON have produced variable results in SLE‐associated ON.^160^ As ON typically affects younger adults, as compared to osteoarthritis, the preservation of the hip joint and the early treatment are equally important for preventing the progression to collapse. Therefore, the interventions including core decompression, stem cell therapy, osteotomies, and non‐vascularized or vascularized bone grafting have been selected in the early stages of ON, with total joint replacement being reserved for end stage disease.

##### Total joint arthroplasty

Total hip arthroplasty is often seen as the last resort and the most aggressive treatment of ON, typically reserved for late‐stage ON when cortical collapse seems imminent. Despite the advanced stage of the disease, the outcomes of this procedure have been fairly positive. Among SLE patients, the annual numbers of total hip joints arthroplasties, partial hip joints arthroplasties, and total knee arthroplasties have shown a statistically significant increase over time.^161^ Figgie et al reviewed the population‐based rate of joint replacement in SLE patients from 1991 to 2005 and demonstrated that the rate of arthroplasty in patients with SLE has been increased from 17% in 1991 to 38% in 2005.^162^ However, the rate of THA for ON has been decreased from 53% in 1991 to 24% in 2005.^162^ Chen et al used The PearlDiver patient records database and reported a 190% of increase in THA for ON in patients with SLE from 2007 to 2015.^163^ THA plays a particularly important role in the treatment of ON in patients with SLE as these patients progress to late stages much more rapidly than other etiologies of ON. Musculoskeletal pain and function are of great concern for these patients.

##### Core decompression

Core decompression (CD), the most frequently used procedure, preserves the structure of the hip and relieves bone pain.^164^ It can reduce intraosseous pressure, penetrate area hardened by fibrotic changes, promote the growth of blood vessels along the tunnel into the femoral head, enhance the formation of new bone, and delay ON.^165^ CD is suitable for early‐ stage ON patients.^166^, ^167^ In addition, CD can provide better clinical or imaging results.^164^ However, there is an element of selection bias in these results, given that the majority of patients receive CD at early stages of the disease.^164^ An additional long‐term study suggested that CD can be an effective alternative treatment for an early stage of glucocorticoid‐associated ON in SLE patients, especially relieving pain and postponing progression to THA.^168^ During the past decade, the efficacy of CD has been improved and techniques such as single large‐diameter drilling and multiple small‐diameter drilling with and without bone grafting have been developed.^169^ It has been suggested that CD plus autologous bone therapy or cytotherapy is a better way to reduce the failure rate of conservative treatment in patients with early and mid‐stage ON.^170^, ^171^

##### Stem cell treatment

The application of stem cell treatment, which many previously considered experimental,^172^ has gained accumulating evidence for clinical improvement.^173^, ^174^, ^175^ A 30‐year follow‐up prospective randomized study based on 125 consecutive patients, bone marrow cell transplantation can be an effective treatment for early‐stage femoral head ON. Bone marrow cell transplantation can delay the progression of the disease, reduce the incidence of collapse, and avoid joint replacement.^176^ The study also found that after excluding some factors that may affect clinical and radiological results, CD and bone marrow mesenchymal stem cells (BM‐MSCs) implantation was an effective method to reduce the THA conversion rate of ON patients, especially for the early‐stage patients. However, CD and BM‐MSCs did not prevent the progress of ARCO staging.^176^ A recent systematic review and meta‐analysis also found that compared with CD treatment alone, the use of MSCs in early stages of ON patients lowered the rates of disease progression and failure and led to fewer minor complications.^177^, ^178^ Implantation of ex‐vivo expanded BM‐MSCs, in combination with CD, has shown promise as a treatment for ON.^179^ In a study conducted by Mardones et al, five ON patients received ex‐vivo expanded MSCs and the hip function of all patients significantly improved. Concentrated autologous bone marrow aspirate transplantation (CABMAT) slows the progression of ON to THA with only minor side effects.^180^

The beneficial effect of stem‐cell therapy has also been shown in the SLE patient population as well. Yoshikoga et al demonstrated that CABMAT significantly improved pain and Harris Hip Scores in eight of nine hips from 18 ON patients with SLE.^181^ Mid‐term follow up for CABMAT showed that ON patients with SLE who received CABMAT had lower conversion rates to THA.^182^ A recent case report using autologous bone marrow aspirate concentrate (BMAC) injections to treat an 18‐year‐old female SLE patient with glucocorticoid‐related ON in bilateral knees showed that after 24 months follow‐up, the patient had improved in function and had pain relief.^183^ Bone marrow aspirate transplantation has the advantages of minimal invasiveness, low cost, simplicity, and the ability to be used in combination to augment other treatment methods as well.^173^

##### Osteotomies

Transtrochanteric rotational osteotomy, an osteotomy procedure predominantly performed in Asia, shifts the weight‐bearing area to a field of healthy bone, relieving the pressure of weight on necrotic bone.^184^ Studies show that the 5‐year and 10‐year hip survival rate of ON patients after transtrochanteric rotational osteotomy is satisfactory in both Asian patients and non‐Asian populations.^184^ After proper selection of patients, accurate surgical procedures, and appropriate postoperative rehabilitation treatment, transtrochanteric rotational osteotomy can be used as an effective hip protection measure for young patients, people with active symptomatic ON, and ON patients with SLE^185^ ^,^.^186^

##### Vascularized and non‐vascularized bone grafting

Bone‐grafting, most commonly as an autogenous vascularized bone or vascularized bone harvested from the fibula or iliac crest, has also been described in the literature as a treatment method for ON.^187^, ^188^ Both vascularized and non‐vascularized bone grafts have been shown to improve outcomes for ON patients by improving joint function and delaying joint repair surgery.^189^, ^190^ A recent systematic review of 15 studies demonstrated that compared with core decompression and non‐vascularized fibular bone grafting, free vascularized fibular transplantation is a better treatment option, especially in young patients who have early‐stage ON, before collapse.^189^ Eighty hips belonging to 50 SLE patients who underwent free vascularized fibular grafting for ONFH were followed for more than 2 years (average 4.3 years) and the hip score improved in all patients.^190^ Numerous studies have shown that a non‐vascularized fibular allograft combined with CD and bone grafting is a cost‐effective way of improving the survival rate for an early stage of ONFH, delaying disease progression, and improving the quality of patients' lives.^191^, ^192^

##### Complication of surgical intervention

Although total joint arthroplasty for SLE patients can generally obtain good or excellent clinical results and improve the quality of life, SLE patients receiving total joint arthroplasty have a higher complication rate than non‐SLE counterparts, which requires various measures to prevent.^193^, ^194^, ^195^ Both SLE and long‐term glucocorticoid use increase the risk of perioperative complications such as wound infection.^160^ Lin et al showed a dose‐dependent relationship between preoperative GC treatment and postoperative complications and mortality in SLE patients.^196^ In patients with SLE, disease activity and infection are the two main causes of death postoperatively.^197^ However, the recent studies showed a lower infection rate, which is likely due to careful patient selection and increased provider precautions.^160^, ^163^, ^193^ Although the use of GCs in SLE patients may increase the risk of wound infection, proper care can improve the infection rate. Another consideration is thrombophilia or hypercoagulability, which are often prevalent in SLE patients. In addition, the prevalence of antiphospholipid syndrome is increased from 1% to 5% in healthy individuals to approximately one third of SLE patients.^198^ The adequate use of prophylactic anticoagulation following orthopedic surgery may diminish the incidence of thromboembolic complications.^199^ It is also crucial to consider that ON patients typically have these components implanted at a younger age, meaning that they must retain these implants for much longer than their non‐autoimmune counterparts. The risks of implant failure and other complications compound as a result.^200^ The complication rate reported in the literature varies greatly, making it difficult to ascertain the true risk of post‐THA surgical complications in the SLE population.

### 5.4 Potential candidates for the management of ON

#### 5.4.1 Hyperbaric oxygen therapy

Hyperbaric oxygen (HBO) therapy increases the level of tissue oxygenation,^201^ which can, in turn, lead to promoting fibroblast proliferation, collagen synthesis, and angiogenesis.^202^ Several studies have suggested that HBO therapy improves the symptom of ON by potentially lowering intraosseous pressure within the femoral head and by improving microcirculation.^203^ Bosco et al showed that HBO therapy can diminish inflammatory cytokines and ROS in patients with ON.^204^ HBO therapy has been suggested to be an effective option for patients with early‐stage ON.^205^ A recent meta‐analysis for 10 studies using HBO therapy as the treatment for ON has identified a significant clinical benefit of HBO therapy on ON in Asian populations.^206^ It is necessary to clarify the effect of hyperbaric oxygen therapy on glucocorticoid‐induced ON in the future.

#### 5.4.2 Natural compounds

The potential efficacy of several natural compounds on ON has been suggested. These natural compounds have a modulatory effect on bone cells – promoting bone formation and inhibiting bone resorption. Genistein aglycone, an isoflavone widely found in soybeans and seen as a natural alternative to selective estrogen receptor modulators,^207^ protected mice from both ovariectomy‐induced bone loss and glucocorticoid‐induced bone loss^208^, ^209^, ^210^ and showed a protective effect on bone loss in postmenopausal women.^211^, ^212^ Bitto et al also found that, in some cases, genistein aglycone also showed a positive outcome for methylprednisolone‐induced necrotic deterioration of the femoral head.^209^ Vinpocetine, a natural compound extracted from the leaves of *Phyllostachys pubescens*,^213^ has also shown a protective role in ON of the femoral head in rat models.^214^ However, the protective effect of natural compounds on ON has not been tested in clinical human studies and needs to be further studied in ON patients.

## 6 CONCLUSION

Osteonecrosis is a complication that can often cause joint pain and loss of function within the joints leading to physical disability for many SLE patients. SLE patients with ON do not typically respond to conservative treatment and eventually require joint replacement. However, the pathogenesis of ON in SLE patients is still controversial. It is important to note that ON develops only in a subset of SLE patients who received high‐dose glucocorticoids. This discrepancy suggests that there are underlying patient‐specific factors that govern susceptibility to ON in the setting of high‐dose GCs. It remains unclear what patient‐specific factor is associated with the incidence of ON in SLE patients. As the underlying reason for susceptibility of ON in SLE patients remains unclear, it is imperative to identify the risk factors that precipitate ON in SLE patients. As shown in Table 1, a high glucocorticoid dosage is a strong association factor for the incidence of ON in SLE patients. Thus, many studies suggested that lowering the dose of oral glucocorticoids can minimize the incidence of ON in SLE patients. However, Chen *et al* demonstrated that SLE patients who received low‐dose glucocorticoid therapy showed a higher risk of GC‐related damages than SLE patients who did not receive GC therapy.^113^ Therefore, identifying a safe dose for preventing GC‐related damage would be critical. As there is no cure for SLE patients and glucocorticoid therapy is important for the management of SLE, identifying the lowest effective doses of glucocorticoids in combination with other agents will be required to minimize the incidence of ON.

Multifocal ON is often found in SLE patients with ON and is characterized by the involvement of multiple separate anatomic sites. It has been suggested that there is a strong relationship between multifocal disease and glucocorticoid therapy. However, it is difficult to interpret the potential effect of glucocorticoid use because of the many variables, including dosage, duration of treatment, and route of administration. Between 80% and 90% of patients tested with multifocal ON had hypofibrinolysis or thrombophilia or both. Because of the high incidence of coagulation disorders in patients with ON, it is difficult to evaluate the difference between patients with multifocal ON and those with less musculoskeletal involvement. It is necessary to conduct epidemiological studies to clarify the etiologic factors of multifocal ON.

High‐dose glucocorticoids are a key risk factor for ON in SLE patients. The death of bone cells is evident in ON bone and the biologic responses to glucocorticoids in bone cells have been extensively studied. The dose and duration of glucocorticoids are significantly associated with the likelihood of developing ON. However, the responses to glucocorticoids in ON patients can be altered by the differences between individuals in glucocorticoid sensitivity, which are influenced by multiple factors including genetic predisposition, metabolic factors, and other factors affecting blood supply.^14^ Therefore, systematic evaluation of the risk factors in ON patients is warranted for prevention and interventions.

The majority of ON patients are in the middle‐aged population who are physically active. Thus, it is critical to improve pain and the function of affected joints and to delay total joint replacement surgery. Recent advances in stem‐cell therapy allow its usage in many orthopedic procedures.^215^ MSC‐based therapy demonstrates promising benefits in animal models, but its therapeutic application is currently limited due to a lack of understanding of the tissue regenerative function of human MSCs in vivo. Autologous MSCs from healthy donors appear to exhibit immunomodulatory and tissue‐protective effects after transplantation.^216^ In contrast, autologous MSCs from SLE patients do not carry immunosuppressive properties.^217^, ^218^ In addition, osteogenesis from the bone marrow aspirate of SLE patients is significantly impaired compared to healthy donors.^219^ Thus, it is crucial to develop a systematic approach to determine whether or not the BM‐MSCs of SLE patients with ON still have normal osteogenic potential.^220^

While arthroplasty is indicated for advanced‐stage ON, the described non‐surgical and surgical interventions have been shown to be effective in the early stages of ON. It has also been suggested that early detection of ONFH in the pre‐collapse stage is associated with an improved and more favorable clinical outcome. Thus, awareness of the need for prevention of glucocorticoid‐induced ON in the early stage is increasing and advanced techniques and interventions to detect ON early in SLE patients under glucocorticoid therapy are required.

## AUTHOR CONTRIBUTIONS

K.K., H.C., and K.‐H.P.‐M. were associated with study conceptualization; K.K., H.C., M.K., and I.S. wrote and prepared the original draft of the manuscript. E.S. and K.‐H.P.‐M. reviewed and edited the final manuscript. K.‐H.P.‐M. acquired funding. All authors have read and agreed to the published version of the manuscript.

## CONFLICT OF INTEREST

The authors declare no conflict of interest.
